# Research Progress and Prospect of Substrate Alternatives for Edible Fungi Based on the “Cycle Production of Plants, Animals, and Fungi”

**DOI:** 10.3390/jof11110790

**Published:** 2025-11-05

**Authors:** Hao-Ran Dong, Ning Jiang, Dan Zhang, Yu Li, Feng Zhou, Zheng-Peng Li, Qiao-Zhen Li, Qi Tan, Mei-Yan Zhang, Hai-Long Yu

**Affiliations:** 1 Institute of Edible Fungi, Shanghai Academy of Agricultural Sciences, National Engineering Research Center of Edible Fungi, Shanghai 201403, China; 20180502@saas.sh.cn (H.-R.D.); jiangning@saas.sh.cn (N.J.); zhangdan@saas.sh.cn (D.Z.); pega@163.com (Y.L.); feng5412@126.com (F.Z.); lizp_ln@126.com (Z.-P.L.); liqiaozhen@saas.sh.cn (Q.-Z.L.); syj0@saas.sh.cn (Q.T.); 2 Engineering Research Centre of Chinese Ministry of Education for Edible and Medicinal Fungi, Jilin Agricultural University, Changchun 130118, China

**Keywords:** edible fungi, alternative substrates, cycle production theory, optimization strategies, future directions

## Abstract

Against the backdrop of global food security and circular agriculture development, edible fungi, as a high-protein food source, have both ecological and economic value in their production model using agricultural and forestry wastes. Based on the “Cycle Production of Plants, Animals, and Fungi” theory, this paper systematically reviews the research progress of alternative substrates for edible fungi. First, alternative substrates are categorized into plant-derived, animal-derived, and microbial-derived types according to their sources. The physicochemical properties, application status, and bottlenecks of each type are analyzed, such as difficult lignin degradation in plant-derived substrates, pollutant risks in animal-derived substrates, and lack of unified application standards for microbial-derived substrates. Second, the mechanisms of key influencing factors including substrate nutritional content, pH and moisture content are elaborated. Furthermore, the paper points out current industrial challenges such as regional resource heterogeneity, difficult control of pretreatment parameters, pollutant residues, and poor batch stability, and summarizes targeted optimization strategies, including regional substrate formulations, precise pretreatment technologies, nutritional regulation, and circular utilization models. Finally, future directions are prospected from four aspects: localized resource utilization, technological innovation, circular model upgrading, and standardized governance, providing theoretical support for the large-scale and sustainable development of the edible fungi industry and contributing to agricultural waste resource utilization and the achievement of “dual carbon” goals.

## 1. Introduction

Against the backdrop of growing global concerns over food security and nutritional health, the value of edible fungi as a high-protein food source is becoming increasingly prominent. The protein content of dried edible fungi ranges from 19% to 35% [1,2], a level comparable to that of livestock and poultry products such as meat, milk, and eggs [3,4], underscoring their excellent potential as a source of high-quality protein. With the global population growing and dietary structures evolving, the demand for protein continues to surge. Mushroom protein, as an alternative protein source, offers promising solutions to these challenges by providing an efficient, nutritious, and eco-friendly option for global diets [4,5]. This transformative shift—specifically in the production of edible fungi—yields remarkable environmental benefits: a 65% reduction in carbon emissions, a 75% decrease in water usage, and an 87% reduction in land usage [6]. These compelling metrics underscore the immense potential of edible fungi cultivation to meet global protein demands. What further distinguishes this approach is its unique ability to utilize agricultural and forestry waste as feedstock, thereby forging an efficient, resource-recycling pathway for protein production that aligns with circular economy principles.

It is estimated that approximately 200 billion tons of organic matter are produced annually from photosynthesis and other agro-industrial activities, presenting a significant environmental hazard. Moreover, the burning, neglect, or monsoon-induced decomposition of such waste further exacerbates environmental degradation [7]. China, as a leading global agricultural producer, generates an estimated 855 million tons of crop straw and 3.7 billion tons of livestock manure annually [8,9,10,11,12,13]. The disposal of these agricultural wastes has emerged as a pressing challenge. Traditional composting techniques are plagued by low lignocellulose degradation efficiency, coupled with the persistence of pathogenic bacteria and antibiotic resistance genes, rendering safe and efficient utilization difficult. In contrast, edible fungi demonstrate unique advantages in converting agricultural wastes into valuable resources, enabling efficient material recycling. For example, cultivating *Stropharia rugosoannulata* typically consumes 20 tons of straw per mu (approximately 0.067 hectares), under such cultivation conditions, high yields can be achieved while benefiting soil health [14]. During growth, edible fungus mycelia secrete extracellular enzymes (e.g., laccases, xylanases) that efficiently break down agricultural wastes into nutrients required for fungal development [15,16,17,18,19]. Livestock and poultry manure also serves as a critical substrate component for edible fungi cultivation [20]. When blended with agricultural wastes (e.g., corn straw, rice straw, wheat straw, waste tea leaves) in optimized proportions and subjected to microbial fermentation, the mixture is transformed into a nutrient-rich substrate that supports the growth of high-quality edible fungi [21,22,23,24]. This innovative approach not only addresses the challenge of agricultural waste management—substantially mitigating pollution from waste accumulation and incineration—but also enables efficient nutrient recycling through edible fungi cultivation. As a result, it generates edible mushroom products with significant economic value, establishing a self-sustaining and economically viable cycle.

China has consistently prioritized the construction of an ecological civilization and proposed the scientifically grounded concept of “Cycle Production of Plants, Animals, and Fungi”. This model effectively integrates the principles of material cycling with modern agricultural development, offering a distinctively Chinese approach to overcoming agricultural resource constraints and advancing sustainable development [25]. “Cycle Production of Plants, Animals, and Fungi” theory extends beyond the traditional “plant-animal” two-dimensional framework by incorporating fungi as an independent ecological component within the agricultural system. Specifically, fungi act as decomposers that secrete extracellular enzymes to break down complex plant and animal residues into smaller, plant-absorbable molecules, thereby completing a closed-loop material cycle where agricultural by-products and residues are converted into valuable, reusable resources [26]. This integration aligns with the 3R principles of circular economy [27] (reduction, reuse, and recycling), achieving synergistic enhancement of both ecological benefits [6,28] (e.g., reduced pollution) and economic returns [29] (e.g., value-added mushroom production).

**Figure 1:**
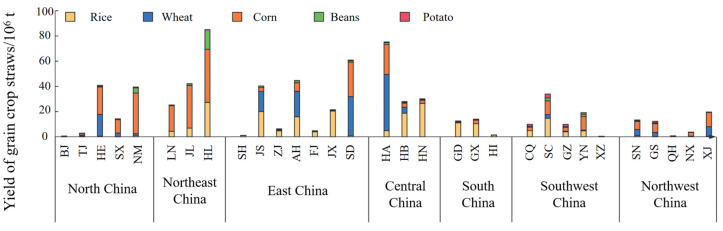
Spatial distribution of different types of crop straw in 2022.

However, despite these advantages, the large-scale implementation of edible fungi cultivation integrated with agricultural waste recycling faces multiple challenges. First, geographical disparities lead to significant variations in both the types and quantities of agricultural residues across different regions [9,30,31], Figure 1 presents the spatial distribution of crop straw in China in 2022. Meanwhile, China’s edible mushroom industry faces challenges such as low industrial agglomeration levels, weak specialization, and pronounced spatial heterogeneity [32], which collectively hinder the standardization of cultivation practices. For example, northern regions primarily use crop straw as substrate while the southwest relies on forestry waste, and regional differences in temperature and humidity further complicate uniform operations. Such variability not only limits scalability but also underscores the critical need for region-specific cultivation protocols and adaptive technologies [33]. Indeed, leveraging local resource endowments and environmental contexts to develop these targeted solutions is pivotal for overcoming standardization barriers, accelerating scalability, and driving high-quality industry development. Notably, region-specific challenges like substrate diversity and environmental adaptation essentially manifest the spatial heterogeneity of cultivation, and the resulting fragmentation of technical solutions further impedes large-scale adoption. Second, the pretreatment of the substrate—which serves as a pivotal step in substrate preparation—demands precise regulation of environmental parameters (temperature, humidity, and pH). Inadequate control of these factors can compromise substrate quality, thereby diminishing both the yield and quality of edible mushrooms [22].

In response to these multifaceted challenges, current research has prioritized two complementary technical pathways: (1) region-specific optimization of cultivation protocols to address spatial heterogeneity, and (2) refined control of substrate pretreatment and environmental parameters to ensure efficient waste utilization. First, researchers are developing localized cultivation frameworks to address technical fragmentation caused by regional substrate diversity and environmental adaptation, including factors like varying straw types and climate-driven variations in humidity and temperature. These include region-specific substrate formulation guidelines, such as adjusting crop straw-to-manure ratios based on local waste composition, including increasing nitrogen in straw-dominated North China or adding carbon sources in forestry waste-heavy Southwest China, and adaptive microbial inoculation strategies, such as selecting spawn strains suited to local temperature or pH conditions. By integrating key local agronomic factors, such as dominant residue types and seasonal climate patterns, into protocol design, these approaches standardize cultivation methods while addressing regional constraints, ultimately reducing the scalability barriers identified in the first challenge. Second, to resolve the critical bottleneck of substrate pretreatment, specifically the need for precise environmental control during microbial fermentation to maintain optimal temperature, humidity, and pH levels, scientists pursue two parallel strategies. During the pretreatment phase, targeted research enhances the physicochemical properties via dynamic parameter optimization, creating an optimal environment for efficient mycelial colonization. Researchers monitor critical indicators substrate particle characteristics [34], pH dynamics [35,36], temperature profiles [35], microbial community succession [23], and changes in the carbon-to-nitrogen (C/N) ratio [37,38] to track how substrate properties evolve during fermentation. This data guides decisions on optimal fermentation duration and endpoint. For broader cultivation, the growth environment is fine-tuned to match diverse waste resources. This includes calibrating oxygen supply by using ventilated tunnel systems for aerated composting or passive aeration in low-resource settings and adjusting humidity levels by modifying misting frequency in high-humidity regions and tailoring light intensity by adopting shaded or open cultivation models based on the specific waste type which includes straw-based or paper pulp-based substrates and local environmental conditions. These strategies directly address inadequate pretreatment control and environmental variability identified earlier, improving substrate quality and fungal yield stability.

This comprehensive review endeavors to systematically synthesize existing research on the utilization of agricultural residues in edible fungi cultivation. By examining the current developmental landscape and key challenges of “Cycle Production of Plants, Animals, and Fungi”, the review aims to propose practical optimization strategies. The pivotal significance of this work lies in providing theoretical support for the large-scale and sustainable expansion of the edible fungi industry, thereby contributing to the harmonious advancement of food security, environmental conservation, and economic prosperity.

## 2. Methods

### 2.1. Data Sources

To comprehensively cover the research progress of edible fungi substrate alternatives based on the “Cycle Production of Plants, Animals, and Fungi” theory, multiple authoritative databases were selected for literature and patent retrieval. For academic literature, English databases including Web of Science Core Collection (WOS), Springer, and ScienceDirect, as well as Chinese databases such as China National Knowledge Infrastructure (CNKI), and Wanfang Data were used. These databases were chosen for their extensive coverage of agricultural science, mycology, environmental science, and circular economy-related research.

### 2.2. Retrieval Keywords

Retrieval keywords were determined based on the core content of the review, including “edible fungi”, “alternative substrate”, “Cycle Production of Plants, Animals, and Fungi”, “agricultural waste”, “lignocellulose”, “livestock manure”, “spent mushroom substrate”, “substrate optimization”, etc. Considering the differences in expression between English and Chinese, corresponding bilingual keywords were designed. For example, English keywords included “edible fungi”, “alternative substrate”, “plant-animal-fungus cycle”, “agricultural waste”, “lignocellulose”, “livestock manure”, “spent mushroom substrate”, “substrate optimization”; Chinese keywords included “食用菌”, “替代基质”, “动植物菌循环”, “农业废弃物”, “木质纤维素”, “畜禽粪便”, “菌渣”, “基质优化”.

### 2.3. Retrieval Time Range

The retrieval time range was set from 1978 to 2025 to cover the historical development and latest progress of the field. After initial retrieval, strict filtering criteria were applied to screen the retrieved results. The percentage distribution of cited literature from different periods is shown in Table 1.

## 3. Classification and Application Status of Alternative Substrate Resources for Edible Fungi

**Figure 2:**
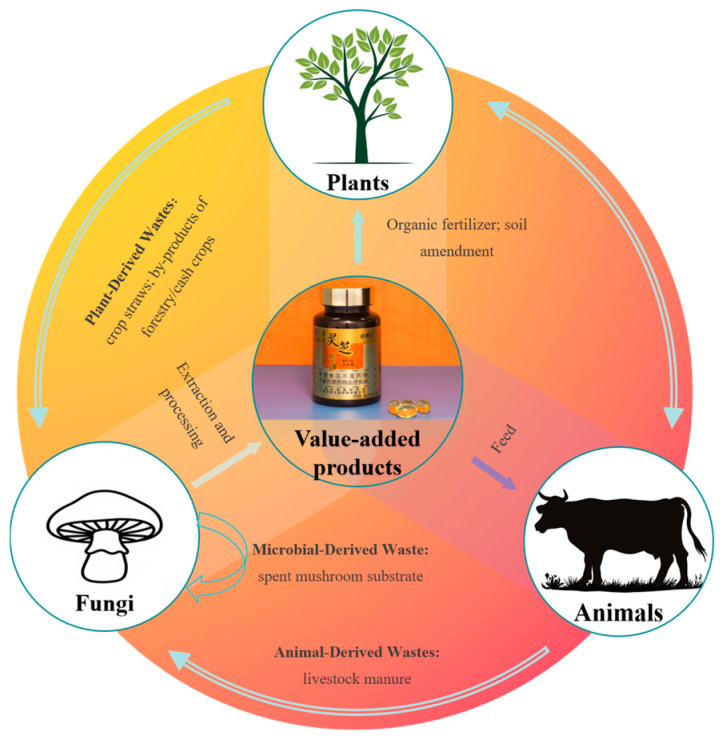
Fungi mediate the conversion of various biowastes into value-added products.

“Cycle Production of Plants, Animals, and Fungi” is a theoretical cornerstone for sustainable agricultural resource utilization, as it integrates the biological synergies of plants, animals, and fungi into a closed-loop system that maximizes resource efficiency and minimizes waste [39]. The core principle of this theory lies in fully leveraging the unique advantages of plants, animals, and microorganisms, and organically integrating their production models to establish a green, efficient, closed-loop agricultural system [40]. Such integration requires a critical operational mechanism: the transformation of diverse biological wastes into value-added products through fungal mediation (Figure 2). Building on this framework, current alternative substrates can be systematically categorized into three types based on origin: plant-derived, animal-derived, and microbial-derived wastes. Despite distinct physicochemical properties, application scenarios, and technical requirements, all these resources operate within the unified “waste resource utilization-fungal transformation -product output” cycle, forming a critical pathway for sustainable agricultural ecosystem development.

### 3.1. Application of Plant-Derived Wastes in Substrate Substitution

As the most abundant and widely distributed agricultural waste resource, plant-derived wastes primarily include crop straws—mainly those from rice, corn, and wheat [41]—and by-products of forestry/cash crops such as branches, fruit shells, sugarcane bagasse [42]. While these materials are ubiquitous, their suitability as fungal substrates varies significantly depending on their lignocellulosic composition, ash content, and physical structure, factors that are often under-addressed in general reviews but critically affect mycelial colonization and fruiting performance. Currently, certain achievements have been made in the application of these wastes as edible fungi substrates, yet several challenges and issues still remain to be addressed.

Despite numerous studies reporting positive outcomes, there is a lack of standardized methodologies for substrate preparation and evaluation, leading to inconsistent data across studies and limiting the broader applicability of reported successes. From a physicochemical perspective, the inherent properties of plant-derived wastes are highly consistent with the nutrient absorption characteristics of edible fungi, and this congruence serves as the core foundation for the application of plant-derived wastes as substrates. Nevertheless, the assumption of inherent “compatibility” overlooks the fact that many plant wastes require proper composting or enzymatic pretreatment to render nutrients bioavailable, a step that is often insufficiently characterized in practice. Specifically, the main components of plant-derived wastes include cellulose, lignocellulosic material, hemicellulose, pectin, and lignin [43,44]. For instance, research data indicates that their cellulose content generally ranges from 40% to 50%, while hemicellulose and lignin account for roughly 20–30% and 10–25%, respectively [45]. Such a constituent makeup establishes a sound carbon-source nutritional foundation for edible fungi mycelia, meeting their core nutrient requirements [46]. However, the relatively high lignin content in some residues (such as sugarcane bagasse) may impede the decomposition process and nutrient release, a factor that is often not differentiated and addressed in relevant literature. Beyond nutrient supply, their loose and porous structure further enhances both air permeability and water-holding capacity. These combined physicochemical properties have been validated through numerous successful practical applications. Yet, inconsistencies in substrate porosity and moisture retention are often observed due to variations in raw material particle size, compaction, and preprocessing methods, which can lead to suboptimal microenvironments for fungal growth. For example, in the cultivation of *Pleurotus ostreatus*, agricultural residues such as rice straw [47], corn stalk [48], corn cob [49], and sugarcane bagasse [50] have been widely used as primary substrates, often achieving high biological efficiency and excellent yield performance. Similarly, in the production of shiitake mushrooms (*Lentinula edodes*), research by López-Balladares has demonstrated that agro-industrial wastes hold significant potential for maintaining both yield and quality of the mushrooms [51]. These plant-derived materials, after proper composting and sterilization, support robust mycelial growth and result in high-quality fruiting bodies rich in bioactive compounds. Field trials in China and other countries have shown that substrates composed primarily of agricultural plant waste can match the performance of traditional wood-based substrates in terms of yield and economic returns [52,53,54], while significantly reducing reliance on forest resources and lowering production costs.

Nonetheless, the long-term effects of using plant-based substrates on mushroom quality attributes, including shelf life, bioactive stability, and heavy metal uptake, remain underexplored, posing potential risks for food safety and market acceptance. However, despite the demonstrated feasibility and economic advantages of plant-derived wastes in edible fungi cultivation, their large-scale practical application remains plagued by significant challenges. A key limitation is the lack of comprehensive substrate formulation databases tailored to specific fungal species and regional feedstock availability, which hinders scalable and replicable applications. These challenges—manifested as sluggish mycelial growth and weakened antioxidant capacity [55], unstable fruiting body yields, and compromised product quality [56]—are fundamentally rooted in nutritional imbalances within the substrates, as well as mismatches between the chemical composition of the waste materials and the species-specific physiological demands of different edible fungal strains [53,55,57]. Notably, edible fungi exhibit differential adaptability to plant-based substrates: *Pleurotus ostreatus* can efficiently utilize both lignin and cellulose in rice straw [58], while *Lentinus edodes* prefers lignin-rich hardwood sawdust [59,60]. This species-specific substrate preference highlights a critical gap in current substrate recommendation systems, which often adopt a “one-size-fits-all” approach despite substantial differences in fungal enzymatic capabilities and nutritional needs. These variations underscore the need for “region-specific substrate formulations”. While the concept of region-specific formulations is conceptually sound, its implementation faces practical hurdles, including the availability of local feedstocks, variability in seasonal raw material properties, and the need for localized composting protocols, all of which require further research and extension support for effective deployment.

### 3.2. Application of Animal-Derived Wastes in Substrate Substitution

Animal-derived wastes, predominantly livestock manure, mainly provide nitrogen (resolving the “nutrient imbalance” issue in pure straw/forestry waste substrates) and micronutrients [61] during edible fungus cultivation. However, the assumption that all types of livestock manure uniformly contribute to balanced nutrition overlooks significant variation in nutrient profiles among poultry, swine, and cattle manures, which can lead to either nitrogen excess or deficiency depending on the source and processing method. They also serve as a critical link between animal husbandry and edible fungus cultivation within the “Cycle Production of Plants, Animals, and Fungi”. Their utilization not only alleviates the nitrogen deficiency common in sawdust/straw but also closes the nutrient loop by converting animal by-products into growth substrates for edible fungi.

However, the challenges regarding the safety and efficiency of animal-derived wastes necessitate rigorous pretreatment protocols. Livestock and poultry manure often contains antibiotics (e.g., quinolones, tetracyclines, and sulfonamides) [62], heavy metals (e.g., Cu, Zn, As, Cr, Cd and Pb) [62], and antibiotic-resistant bacteria (e.g., *Escherichia coli*, *Salmonella*) [63]. The presence of antibiotic residues and resistant genes not only poses direct risks to fungal and human health but may also contribute to the spread of antimicrobial resistance in agroecosystems that is insufficiently addressed in most substrate safety assessments. Globally, up to 73% of all antimicrobials are used in food-producing animals, with 30–90% of these excreted via urine and/or feces [64] due to their low metabolic clearance [63]. Residual antibiotics, metabolites, and antibiotic resistance genes are widespread, with adverse effects on living organisms [65]. To address these multifaceted hazards, a suite of pretreatment technologies has been optimized for compatibility with the “Cycle Production of Plants, Animals, and Fungi” framework, balancing efficacy, cost, and environmental sustainability. Composting remains the most widely adopted method for managing the large volumes of waste generated by the poultry industry. It serves as an effective and sustainable approach that not only reduces associated environmental impacts but also transforms waste into a valuable agricultural resource [66,67]. Yet, traditional composting may not fully degrade persistent antibiotics or eliminate antibiotic resistance genes. Its efficacy is highly dependent on process parameters, which are often not adequately monitored in routine operations. Okada et al. [68] found that the quality of compost is influenced by composting process conditions (such as additional carbon sources and mechanical aeration) and co-substrate selection (for example, treated wood shavings may accumulate potentially toxic elements). This underscores the complexity and variability of composting outcomes, which are often context-specific and poorly standardized, thus limiting reproducibility and broad adoption. Notably, targeted regulation of process conditions can mitigate such variability. A mechanical aeration system without added carbon sources can effectively reduce the content of antibiotic residues, and antibiotic-resistant *E. coli* in poultry litter, while producing mature, stable, and sanitized compost. Co-composting animal-derived waste with carbon-rich rice straw/sawdust optimizes the C/N ratio for microbial activity to boost efficiency and reduces heavy metal bioavailability via complexation with humic substances [69]. Li et al. and Serramiá et al. noted that animal manure lowers the high C/N ratio of lignocellulosic materials and accelerates composting [70,71].

Optimizing the C/N ratio is central to supporting the growth of saprophytic fungi, given that an excess of nitrogen can lead to toxic accumulation of ammonia or imbalanced carbon-nitrogen metabolism, thereby preventing mycelial development [72]. While this principle is well-established, the optimal C/N range reported in the literature often reflects controlled experimental conditions and may not translate directly to diverse substrate compositions or environmental contexts encountered in practice. As reported in existing literature, a lower C/N ratio is more conducive to the mycelial growth of oyster mushrooms [73], which in turn contributes to enhanced yield and biological efficiency in oyster mushroom cultivation [74]. Specifically, the optimal C/N ratio range for oyster mushroom cultivation has been identified as 19:1 to 22:1 by relevant studies [74,75]. An excessively high C/N ratio, however, tends to result in insufficient nitrogen content in the substrate. This nitrogen deficiency not only inhibits the growth of mycelia but also leads to a slower spawn running rate. Conversely, an overabundance of nitrogen in the substrate may exert a negative impact on the fruiting body formation process by delaying its development [76].

These findings on C/N ratio optimization are reinforced by practical application outcomes, which highlight significant benefits when animal-derived wastes are used to fine-tune substrate nutrition. For instance, when *Agaricus bisporus* is cultivated on substrates containing 52% decomposed chicken manure, the mushroom yield reaches 20.74 kg·m^−2^, representing a 17% increase compared with the control group [77]. These results underscore the value-added potential of animal-derived wastes in mushroom production.

### 3.3. Application of Microbial-Derived Waste in Substrate Substitution

Spent mushroom substrate (SMS) is a waste residue generated during commercial mushroom cultivation, and its weight is typically as much as five times that of the harvested mushrooms themselves [78,79]. With the rise in the “circular economy” concept, researchers have begun to investigate the potential utilization value of spent mushroom substrate, and the results have shown that it contains substantial untapped value [80], such as for composting, substrate for other mushroom-forming fungi, biomass fuel, animal feed, and material substrates [80,81]. While these reuse options are diverse, their economic feasibility and market demand vary considerably, and many applications remain at the experimental or pilot stage without clear pathways to scalability. Among these applications, reusing SMS as a cultivation substrate for edible mushrooms has become a research hotspot, as its residual nutrients and porous structure are highly compatible with fungal growth—addressing both waste disposal and substrate cost issues simultaneously.

SMS is rich in residual nutrients such as carbohydrates, proteins, and fats, whose presence makes it possible to cultivate mushrooms using it. However, the nutrient composition of SMS depends heavily on the original mushroom species, substrate composition, and cultivation conditions, resulting in inconsistent performance during reuse. Notably, this variability is rarely quantified in studies that assert the efficacy of SMS. Studies have shown that the residues of *Pleurotus ostreatus*, *Pleurotus eryngii*, *Hypsizygus marmoreus*, and *Agaricus bisporus* partially replace waste cotton for the production of *Volvariella volvacea* [82,83]. Marianna Dedousi et al. demonstrated that SMS is beneficial for increasing the intracellular polysaccharide (IPS) content in the fruiting bodies of *Pleurotus ostreatus* and *Pleurotus eryngii*, while the combination of spent mushroom substrate and Pleurotus waste (PW) is conducive to the synthesis of proteins in the fruiting bodies [84]. SMS has demonstrated enormous application potential in the field of edible mushroom cultivation. However, due to the significant differences in physicochemical properties among SMS from different sources, it is difficult to form unified standards for its addition method, form during addition, and addition ratio, which has restricted its large-scale application to a certain extent. For instance, Gonçalves Vieira Junior et al. found that adding SMS exerted a positive effect in the third cultivation cycle, and the optimal results were achieved when 5% to 10% SMS was added under high-nitrogen substrate conditions [85]. Hu et al. introduced a novel application strategy in the mushroom production industry to convert conventional SMS into renewable biochar-based SMS. They evaluated the application benefits of this renewable SMS in edible mushroom production through a cultivation experiment with *Pleurotus ostreatus*. The results showed that, compared with conventional SMS, the renewable SMS increased mushroom yields by 20–25%, shortened fruiting time by 4–6 days, and achieved significant economic benefits [86].

To summarize, spent mushroom substrate has emerged as a promising alternative substrate for edible mushroom cultivation, with multiple studies validating its ability to enhance yields, improve nutritional quality, and reduce production costs. Nevertheless, these benefits are often reported in optimized experimental settings and may not hold under real-world farming conditions where substrate mixing, pest pressure, and environmental variability are less controlled. However, the lack of unified standards for its application—stemming from variable physicochemical properties across SMS sources—remains a key barrier to large-scale adoption. Future research should prioritize standardizing SMS quality assessment, optimizing application protocols tailored to different mushroom species and substrate types, and quantifying economic and environmental benefits to fully unlock its potential in circular agriculture. More targeted future research should also focus on three key areas, which remain underdeveloped despite their critical importance. First, it should work on developing rapid, low-cost methods for evaluating SMS quality; second, it needs to assess the long-term impacts of SMS reuse on soil health and fungal productivity; third, it should integrate SMS utilization into policy frameworks for sustainable agriculture.

## 4. The Key Influencing Factors and Mechanisms of Substrate Substitution

**Figure 3:**
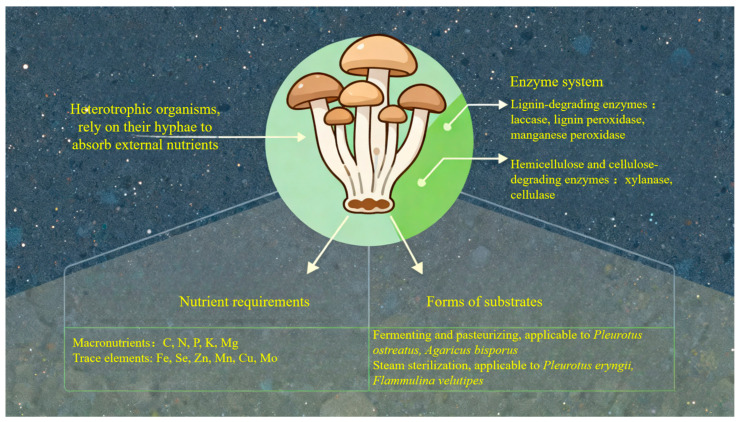
Elements in mushroom cultivation: nutrition, enzymes, and substrates.

Unlike plants, mushrooms are heterotrophic organisms that rely on their hyphae to absorb external nutrients for their own growth [87]. Mushrooms produce a variety of enzymes that facilitate the degradation of lignocellulosic substrates [28,88,89], mainly including lignin-degrading enzymes (laccase, lignin peroxidase, manganese peroxidase) as well as hemicellulose and cellulose-degrading enzymes (xylanase, cellulase). Additionally, mushrooms need oxygen and a specific pH level to maintain normal metabolism and grow well. Carbon (C) and nitrogen (N) are the two key macronutrients fungi require for building structures and producing energy. Phosphorus (P), potassium (K), and magnesium (Mg) are also considered macronutrients for mushrooms. Furthermore, trace elements like iron (Fe), selenium (Se), zinc (Zn), manganese (Mn), copper (Cu), and molybdenum (Mo) seem essential because they play roles in various physiological processes [90]. There are two main forms of substrates used in mushroom cultivation. One is compost materials obtained by fermenting and pasteurizing agricultural wastes, and the other is non-compost materials formed by mixing different agricultural wastes followed by steam sterilization. Such agricultural wastes usually include straw, plant fiber/husk, manure, or sawdust, etc. The compost-formed substrate is used for cultivating *Pleurotus ostreatus* [91] and *Agaricus bisporus* [92], whereas non-compost-formed substrates can be used for cultivating *Pleurotus eryngii* [93], *Flammulina velutipes* [94], and *Auricularia auricula-judae* [95] (Figure 3). Different mushroom species have varying substrate requirements, making the selection and formulation of substrates particularly crucial in the cultivation process. The choice and combination of substrates must take into comprehensive consideration factors such as nutritional content, pH, aeration, and water retention capacity, to ensure the provision of an optimal microenvironment for mycelial colonization.

### 4.1. Nutritional Content

The nutritional content of the substrate significantly affects biological efficiency and quality characteristics of mushrooms [96,97]. Different substrates, due to variations in their nutritional composition, play a regulatory role throughout the entire growth process of mushrooms. Table 1 provides examples of studies on the biological efficiency of various substrates derived from lignocellulosic wastes used in cultivating mushrooms.

The C/N ratio serves as a key parameter for evaluating the suitability of alternative substrates for edible mushroom cultivation. However, different mushroom species exhibit distinct requirements for the substrate C/N ratio. This necessitates careful consideration of the inherent C/N characteristics of agricultural waste materials when using them as substrates. Table 2 presents the C/N ratios of common agricultural waste materials. Specifically, appropriate combinations and formulations must be designed to create a substrate with an optimal C/N ratio tailored to the target edible mushroom, thereby ensuring the highest yield can be achieved within the shortest possible cultivation period [98]. Formulating substrates with precise C/N ratios is often hindered by the lack of affordable, real-time analytical tools for on-farm nutrient assessment, limiting the practical application of these recommendations in smallholder or resource-limited settings. According to Hu’s research, adding 15% biochar to the *Pleurotus ostreatus* substrate significantly reduced the mycelial colonization rate, and resulted in total crop failure during the second flush [86]. The main reason may be that the excessive addition of carbon sources led to an excessively high C/N ratio in the substrate, which exceeded the optimal growth range for *Pleurotus ostreatus*, thereby causing inhibition [99]. To mitigate the risk of growth inhibition caused by C/N ratio imbalance, careful formulation of the substrate is essential. In addition to limiting the inclusion of high-carbon materials such as biochar, incorporating nitrogen-rich additives—including rice bran, soybean meal, or poultry manure—can effectively balance the overall C/N ratio within the optimal range for *Pleurotus ostreatus* (commonly 20:1 to 30:1). However, adding nitrogen-rich materials comes with the risk of ammonia accumulation. This is particularly true in conditions with high temperatures or poor aeration, as such ammonia buildup can be just as harmful to both mycelial growth and the development of fruiting bodies. So, this situation demands balanced management. Furthermore, pre-treatment methods such as composting or fermentation can help stabilize the C/N ratio [100], enhance nutrient availability, and improve substrate structure, thereby creating a more favorable environment for consistent mycelial growth and reliable fruiting across multiple flushes.

In addition to the C/N ratio, mineral nutrients in the substrate are equally crucial for supporting the metabolic activities, growth, and quality formation of edible mushrooms. Although minerals are essential, applying them excessively can cause antagonistic interactions, toxicity, or imbalances. These problems can suppress mycelial vigor or fruiting. Yet, this risk is often not given enough attention in substrate formulation. These inorganic elements are involved in enzyme activation [106], inducing gene transcription and expression [107], and directly influence mycelial vigor, fruiting body development, and resistance to environmental stresses. Generally, lignocellulosic materials themselves have a low mineral content, so minerals need to be added to provide various essential nutrients, thereby enhancing mushroom yield [108]. In the culture medium, minerals such as phosphorus, magnesium, sulfur, calcium, iron, potassium, copper, zinc, manganese, and cobalt are commonly added according to the specific nutritional requirements of edible fungi, to ensure proper mycelial germination and normal fruiting body formation. For instance, the study by Yang et al. showed that iron, zin, and calcium ions are enriched in the mycelia of *Lentinula edodes*, *Flammulina velutipes*, and *Pleurotus eryngii*. At low concentrations, these ions all exhibit promotional effects on mycelial growth, although the optimal concentrations that promote growth vary among different mushroom species [109]. Nakakubo et al. demonstrated that the absence of potassium in the growth substrate significantly prolongs the cultivation period of *Pleurotus ostreatus*, while also reducing the number of primordia, overall yield, and product quality [110]. Therefore, providing an appropriate balance of mineral nutrients in the substrate is essential for optimizing the growth, yield, and quality of edible mushrooms, and the specific requirements may vary depending on the fungal species. Obtaining this balance right remains a major challenge when formulating substrates, particularly in areas where resources are scarce. Here, access to analytical tools, mineral supplements, and technical know-how is often limited, which, in turn, highlights the urgent need for nutrient management strategies that are easy to use, affordable, and can be scaled up.

### 4.2. pH

The pH value is one of the key environmental factors influencing the growth and development of edible mushrooms. It not only directly affects mycelial growth rate, colonization efficiency, and fruiting body formation but also indirectly impacts the overall efficiency and yield of the cultivation process by regulating enzyme activity, nutrient availability, microbial community structure, and metabolic pathways in the substrate. Different species of edible mushrooms exhibit significant variations in their adaptive range to substrate pH, showing distinct species specificity. For instance, according to Aditya et al.’s summary and review of previous studies, the optimal pH for the mycelial growth of *Pleurotus ostreatus* was reported to be 6–7 [111]. The study by Su et al. showed that when *Coprinus comatus* was cultivated using corn stalks as the substrate, the highest lignin degradation (or lignin consumption) occurred at pH 8 [112]. Moreover, the pH requirements may differ for the same variety at various growth stages. For instance, during the mycelial growth phase of *Lentinula edodes*, the pH is maintained between 5.5 and 6.5, whereas during the fruiting body development stage, the pH ranges between 3 and 4. If the substrate pH deviates from the suitable range, it may lead to issues such as slow mycelial growth, reduced colonization ability, impaired nutrient absorption, or yield reduction [85].

In addition to influencing the macroscopic growth and development of edible mushrooms, pH also plays a pivotal role at the microscopic level, particularly in the nutrient absorption process of mycelia. For instance, the absorption of elements such as manganese, magnesium, and copper is influenced by pH [113]. Copper is essential for inducing laccase transcription and production [106,114]. Specifically, under acidic pH conditions (e.g., pH 4.5–5.5), copper ions remain in soluble, bioavailable forms, allowing efficient uptake by mycelial membrane transporters. This adequate copper supply upregulates the expression of laccase-related genes (e.g., *lac1*, *lac2*), thereby enhancing laccase secretion—an enzyme critical for lignin degradation in the substrate and subsequent nutrient release. Conversely, at alkaline pH (pH > 7.0), copper ions tend to form insoluble hydroxide precipitates, reducing their absorption by mycelia. This deficiency not only inhibits laccase synthesis but also impairs the mycelium’s ability to break down complex organic matter, creating a cascade effect that limits the breakdown of lignocellulosic carbon and the release of bound nitrogen, thereby restricting carbon and nitrogen acquisition. This cascade highlights the interconnectedness of pH, enzyme function, and carbon/nitrogen cycling, yet practical strategies to buffer or dynamically adjust pH during cultivation remain underdeveloped and deserve greater attention.

### 4.3. Moisture Content

Water is widely regarded as one of the most critical abiotic factors for successful edible mushroom cultivation, as it supports nearly all physiological activities—from the early stage of mycelial colonization to the final maturation of fruiting bodies. Its function is not limited to serving as a fundamental structural part of fungal cells; in fact, water makes up 80–90% of the fresh biomass of both mycelia and fruiting bodies. Macronutrients and micronutrients such as nitrogen, phosphorus, and potassium are absorbed by the mycelium along with water. Water serves both as a physical medium and a nutrient carrier, and this dual role makes water management in substrates more complex than it seems. Yet the way substrate moisture, nutrient mobility, and mycelial uptake efficiency work together has not been studied enough, particularly when it comes to field-based or low-input cultivation. When agricultural waste is used as a substrate for edible mushrooms, its water—holding capacity varies due to its inherent structural characteristics, particle size, matrix proportioning and processing method [59,102,115]. For example, wheat straw has a stronger water-holding capacity than spent *Pleurotus ostreatus* substrate [102]. The moisture content of the substrate with a mass ratio of 60 beech wood-chip–20 wheat straw–20 wheat bran was significantly higher than that of the substrate with a mass ratio of 75 hazelnut husk–15 beech wood-chip–10 wheat bran; the former had a moisture content as high as 69.94%, while the latter was only 61.93% [59]. These comparative data are valuable but may not reflect real-world performance, as substrate water retention is also influenced by compaction, aeration, and ambient humidity. These factors are variables that are not always controlled or reported in such studies. However, excessively high moisture content in the substrate is not beneficial. Research indicates that overly moist substrates can impede mycelial respiration, disrupt metabolic processes, hinder fruiting body formation, and increase risks of bacterial contamination and nematode infestation [72]. Similarly, insufficient substrate moisture content is equally detrimental to edible mushroom production. Academic studies demonstrate that *Hericium erinaceus* mycelia exhibit significantly lower activities of β-D-glucosidase, cellobiohydrolase, Mn-peroxidase, and laccase at harvest stage under low substrate moisture conditions compared to higher moisture treatment groups (*p* < 0.05) [116]. Although reduced enzyme activity under low moisture is concerning, the extent to which these enzymatic changes directly translate into lower yield or quality remains unclear, as compensatory mechanisms or shifts in metabolic pathways may partially offset the impact.

## 5. Challenges and Optimization Strategies

**Figure 4:**
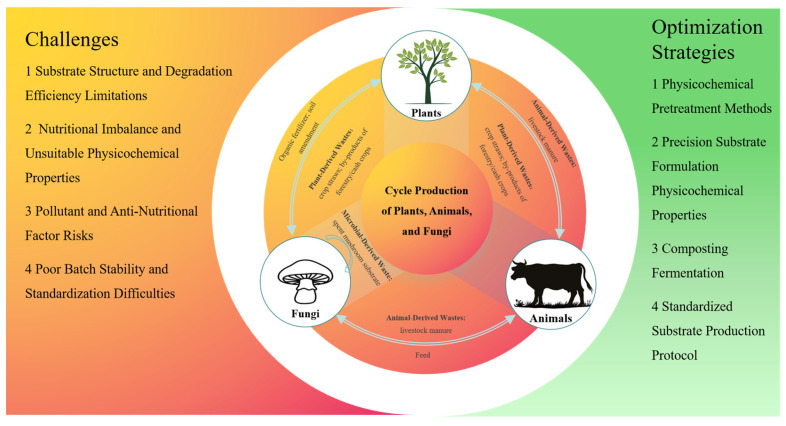
Challenges and optimization strategies.

Under the guidance of the “Cycle Production of Plants, Animals, and Fungi” theory, large-scale cultivation of edible fungi using agricultural waste as alternative substrates provides an effective path for recycling and low-carbon agricultural development. However, it still faces multiple practical challenges. These challenges are concentrated in four aspects: low degradation efficiency caused by the structural limitations of the substrate itself, nutritional imbalance and unsuitable physicochemical properties resulting from the inherent characteristics of wastes, pollutant and anti-nutritional factor risks carried by substrates such as livestock manure, and poor batch stability and standardization difficulties due to composition fluctuations of wastes from different sources. These issues collectively restrict the efficient utilization of substrates by fungi and affect the stability of edible fungi yield and quality. In response to the above bottlenecks, existing research has developed a set of multi-dimensional optimization strategies. These strategies break down substrate structural barriers through physical and chemical pretreatment, balance substrate nutrients via precise proportioning and nutrient supplementation, eliminate safety hazards through targeted treatment, and improve industrial efficiency by combining circular utilization models and standardized processes, providing technical support for promoting the large-scale and sustainable development of the edible fungi industry (Figure 4).

### 5.1. Substrate Structure and Degradation Efficiency Limitations

Agricultural wastes, predominantly composed of lignocellulose, pose inherent structural barriers that severely restrict their biodegradability by edible fungi, directly impairing mycelial colonization and fruiting body formation. The core challenge lies in the recalcitrant nature of lignin, which forms a protective matrix around cellulose and hemicellulose, preventing fungal hydrolytic enzymes (e.g., cellulase, xylanase) from accessing their substrates [117]. For instance, agave bagasse—a major byproduct of mezcal production with annual generation exceeding 480,000 tons in Mexico—contains 41–44% cellulose, 19–22% hemicellulose, and 15–16% lignin [118]. In its raw state, this lignin-cellulose complex resists degradation by *Pleurotus ostreatus* and *Lentinula edodes*, leading to a 20–30% reduction in mycelial growth rate compared to pretreated substrates. Similarly, hazelnut husk, a potential substrate for *Lentinula edodes*, has a lignin content of 22–25% [59], which delays mycelial colonization by 3–7 days and reduces biological efficiency by 15–20% when used without modification. Woody agricultural wastes, such as sawdust and beech wood shavings, present even greater challenges due to their high lignin content (10–25%) and dense fibrous structure. *Pleurotus eryngii* grown on untreated sawdust exhibits uneven mycelial spread, with only 60–70% substrate colonization after 21 days, whereas wheat straw—with a lower lignin content (5–8%)—achieves full colonization in 14–16 days [105]. These examples clearly illustrate how substrate composition (especially lignin content) and structural properties (e.g., fiber density, particle size) collectively influence fungal degradation efficiency and subsequent cultivation performance. For a comprehensive overview of how different substrate compositions, coupled with various pretreatment strategies, impact mushroom yield across multiple studies, refer to Table 3. Comparative analysis of substrate composition, pretreatment methods, and yield performance in mushroom cultivation studies. This structural resistance is further exacerbated by particle size: large, uncrushed corncobs (particle size > 5 cm) retain compact cellulose bundles that limit enzyme penetration, resulting in a 25% lower degradation rate than crushed corncobs [34]. Additionally, the crystalline structure of cellulose in agricultural wastes reduces its accessibility to fungal enzymes. Rice straw, for example, has a cellulose crystallinity index of 45–50%, which inhibits cellulase activity by restricting enzyme-substrate binding [75]. Even when fungi secrete lignin-degrading enzymes like laccase, the low porosity of raw substrates (e.g., intact wheat straw has a porosity of <30%) limits enzyme diffusion, further hampering lignocellulose breakdown [108]. These structural limitations not only prolong the cultivation cycle but also reduce nutrient utilization efficiency. *Pleurotus ostreatus* grown on raw date palm leaves—with a lignin content of 18–20%—exhibits a 12–15% lower biological efficiency than when grown on date palm leaves mixed with wheat straw (25:75 ratio), as the latter’s lower lignin content (8–10%) enhances enzyme accessibility [108]. Similarly, raw coffee residue, with its high lignin (14–16%) and phenolic compound content, suppresses laccase activity by 30–40% in *Pleurotus sajor-caju*, further slowing lignin degradation [97].

In summary, the complex lignocellulosic structure of agricultural wastes—characterized by high lignin content, dense fiber packing, and cellulose crystallinity—creates a multi-layered barrier to fungal degradation. This not only delays mycelial growth and substrate colonization but also limits the overall yield and quality of edible mushrooms, making pretreatment an indispensable step for efficient substrate utilization.

### 5.2. Nutritional Imbalance and Unsuitable Physicochemical Properties

A critical bottleneck in using agricultural wastes for edible mushroom cultivation lies in their inherent nutritional imbalance and unfavorable physicochemical properties, which directly disrupt fungal growth metabolism and substrate microecology. The most prominent issue is the imbalance of C/N ratio—a key determinant of mycelial proliferation. Most lignocellulosic wastes are carbon-rich but nitrogen-deficient: rice husk has an extremely high C/N ratio of 80:1 [75], while wheat straw and corn stover range from 50:1 to 70:1 [74], all far exceeding the optimal range (20:1 to 30:1) for edible fungi like *Pleurotus ostreatus* and *Agaricus bisporus*. This nitrogen limitation inhibits the synthesis of fungal hydrolytic enzymes (e.g., cellulase and xylanase), as demonstrated by Arce-Cervantes et al. (2015) [117], who found that mycelial growth rate of *Agaricus bisporus* on C/N-imbalanced wheat straw was reduced by 35% compared to nitrogen-supplemented substrates. Conversely, nitrogen-rich wastes such as fresh poultry litter (C/N ≈ 10:1) often lead to excessive ammonia release during composting, which is toxic to mycelia, causing a 20–25% reduction in colonization rate [68].

Moisture content is another pivotal factor that regulates substrate aeration and microbial activity. Fresh agricultural wastes like fruit pomace and vegetable trimmings have moisture contents as high as 85–95% [101]. When used directly, they form compact, waterlogged substrates with poor oxygen permeability, creating anaerobic conditions that favor the growth of competitive bacteria (e.g., *Bacillus* spp.) and plant-parasitic nematodes. This not only reduces mushroom yield by 15–30% but also increases the risk of diseases like brown rot [105]. In contrast, dried agricultural wastes such as rice straw and sawdust often have moisture contents below 15% [108]. This desiccation limits the solubility and availability of nutrients, as fungal mycelia rely on aqueous environments to absorb soluble sugars and minerals. For example, Pleurotus ostreatus grown on dried date palm leaves (12% moisture) showed a 40% slower colonization rate than leaves rehydrated to 65% moisture [108].

Beyond C/N ratio and moisture, other physicochemical properties of agricultural wastes further exacerbate cultivation challenges. pH imbalance is common: coffee residue has an acidic pH of 4.5–5.0, while poultry manure-based compost is alkaline (pH 8.0–8.5) [68]. Since most edible fungi thrive in neutral to slightly acidic conditions (pH 6.0–6.5), extreme pH values inhibit enzyme activity; for instance, laccase activity in *Pleurotus sajor-caju* was reduced by 28% on coffee residue compared to pH-adjusted substrates [97]. Additionally, high ash content in some wastes (e.g., rice husk ash content of 10–12% [75]) increases substrate bulk density, reducing porosity and gas exchange, which is detrimental to mycelial respiration. The variability in these properties across waste sources further complicates standardized cultivation. For example, wheat straw from different cultivars has crude protein contents ranging from 3.5% to 6.2% [119], leading to inconsistent C/N ratios and, consequently, a 20–30% variation in *Pleurotus ostreatus* yield. Similarly, date palm leaves collected from different regions have moisture contents differing by up to 15% [108], making it difficult to maintain uniform substrate conditions at scale.

These nutritional and physicochemical limitations collectively hinder the efficient utilization of agricultural wastes by edible fungi. Addressing them requires targeted adjustments—such as nitrogen supplementation, moisture regulation, and pH correction—to create a substrate microenvironment conducive to fungal growth and fruiting.

### 5.3. Pollutant and Anti-Nutritional Factor Risks

Livestock manure, a common agricultural waste used in mushroom cultivation, carries hidden contamination risks. Studies note that it may contain heavy metals like lead and mercury, which can accumulate in edible mushrooms. For example, when *Pleurotus ostreatus* is grown on manure-containing substrates, lead content in fruiting bodies can exceed safe limits if not controlled [101]. Additionally, residual antibiotics from livestock farming persist in manure; improper composting fails to fully degrade them, leading to antibiotic residues in mushrooms that pose health risks to consumers.

On the other hand, some organic-rich wastes hinder fungal metabolism. Coffee grounds, for instance, contain high levels of phenolic compounds. These substances suppress the activity of laccase—a key enzyme for lignin degradation—in fungi like *Pleurotus ostreatus* and *Pleurotus eryngii*, reducing lignin breakdown efficiency by 30% or more [105]. This not only slows mycelial colonization but also limits nutrient release from substrates, ultimately lowering mushroom yield and quality.

### 5.4. Poor Batch Stability and Standardization Difficulties

A major headache for large-scale mushroom farming is the huge variability in agricultural waste composition across sources, which makes it hard to keep production consistent. Take wheat straw, a commonly used substrate—its cellulose content can swing between 35% and 45% just because of different cultivars [119]. For example, straw from high-starch wheat varieties has lower cellulose but higher ash content, while drought-resistant cultivars produce straw with denser fiber structures and higher lignin levels. This inconsistency directly translates to unstable mushroom yields: *Pleurotus ostreatus* grown on these straws can have a yield variation coefficient of 20% to 30%, with some batches hitting 119 g per packet and others dropping to just 49 g [75]. It is not just wheat straw—other wastes show similar fluctuations. Date palm leaves collected from different regions of Saudi Arabia have lignin contents ranging from 18% to 25% [108], and their moisture levels can differ by 10% to 15% depending on harvest season. When used to grow *Pleurotus ostreatus*, this leads to uneven mycelial colonization: leaves with lower lignin fully colonize in 14 days, while lignin-rich ones take 21 days or more. Coffee residue, too, varies widely—its phenolic compound content can jump from 3% to 6% based on coffee bean origin and processing methods, which suppresses laccase activity in *Pleurotus sajor-caju* by 25% to 40% in some batches [97].

This variability makes standardization nearly impossible. Mushroom farms often have to adjust water, supplements, and incubation times for each batch of waste, increasing labor costs and raising the risk of contamination. For instance, if a batch of wheat straw is drier than usual, adding too much water can create anaerobic conditions, while adding too little leaves the substrate nutrient-poor [108]. Such unpredictability is a big barrier to scaling up mushroom production using agricultural wastes.

### 5.5. Optimization Strategies

To address the bottlenecks of agricultural wastes in edible mushroom cultivation, targeted technical optimizations are essential. Table 4 lists the benefits and limitations of different technical optimizations. Alkaline pretreatment stands out for breaking down lignocellulosic barriers—using nejayote, an alkaline effluent from tortilla production, to soak agave bagasse not only reduces furfural content by up to 98% but also boosts lignin degradation rate by 49% and soluble sugar availability by 30–50%, creating ideal conditions for *Pleurotus ostreatus* and *Lentinula edodes* colonization [118]. However, the reliance on nejayote (a tortilla industry byproduct) limits its scalability to regions without maize-based food processing, and the high alkalinity (pH > 11) may require neutralization steps to avoid substrate phytotoxicity for certain mushroom species. Physical pretreatment is equally practical: crushing straw and banana leaf midribs to 2–5 cm fragments enhances heat and moisture retention during composting, and combining this with 121 °C steam sterilization for 30 min accelerates *Pleurotus ostreatus* mycelial growth by 25–40% compared to untreated substrates [75]. Yet, mechanical crushing increases energy consumption (e.g., 2–3 kWh per 100 kg of biomass), and steam sterilization raises operational costs due to fuel/energy demands, which may not be feasible for smallholder farmers in low-resource settings. For woody wastes like hazelnut husk, grinding followed by steam treatment shortens mycelial colonization time by 3–5 days, effectively mitigating the inhibition of high lignin content [59]. Nonetheless, the hardness of woody materials (e.g., hazelnut husk) requires specialized grinding equipment, increasing upfront investment costs, and the efficacy of steam treatment varies with lignin type (e.g., syringyl vs. guaiacyl lignin), limiting universal applicability.

Nutritional regulation through waste blending and supplementation is key to balancing substrate nutrients. Mixing rice straw with 10% poultry litter and 1% lime adjusts the C/N ratio to the optimal 20–30:1 range, increasing *Pleurotus ostreatus* yield to 119 g per packet—42% higher than pure rice straw [75]. However, poultry litter may introduce pathogenic microbes (e.g., *Salmonella*) if not properly composted, necessitating additional safety checks. Moreover, the odor from poultry litter can be a nuisance in indoor cultivation facilities, requiring mitigation measures. Date palm leaves mixed with wheat straw at a 25:75 ratio leverage wheat straw’s balanced nutrients to improve substrate palatability, raising biological efficiency by 18% compared to single-date palm leaf substrates [108]. Yet, the availability of wheat straw is seasonal and geographically constrained (e.g., limited in tropical regions), reducing the reproducibility of this blend in diverse farming systems. Adding corn meal as a supplement significantly enhances laccase activity in *Agaricus bisporus*, promoting lignin decomposition by 22% and increasing cumulative yield to over 34 kg/m^2^ [117]. Response surface methodology further optimizes formulations: a blend of 12% rice bran and 25% food waste compost boosts *Pleurotus ostreatus* yield to 91 g/bottle, 153% higher than the traditional 80% sawdust with 20% rice bran formula [101].

For livestock manure, composting at 60 °C or higher for 7 days degrades over 90% of antibiotic residues, as high temperatures inactivate microbial communities involved in antibiotic persistence [68]. Yet, achieving and maintaining 60 °C uniformly in small-scale compost piles is challenging (e.g., due to poor insulation or irregular turning), leading to incomplete antibiotic degradation in some batches. Additionally, long composting durations (≥7 days) delay substrate availability, impacting cultivation timelines. Additionally, pre-washing coffee grounds to remove excess phenolic compounds reduces their inhibition of laccase activity by 30%, ensuring normal lignin degradation by *Pleurotus sajor-caju* [97].

Circular utilization and process standardization improve economic efficiency and stability. Crushing SMS and mixing it into new substrates at 20–40% replaces part of the fresh lignocellulosic waste, increasing *Pleurotus ostreatus* biological efficiency by 15–20% and cutting raw material costs by 30% [105]. Standardizing substrate preparation—such as controlling moisture at 65–70% via the “hand-press test” (no water runoff when squeezed) and sterilization parameters (121 °C, 1.5 kg/cm^2^ for 1 h)—reduces batch yield variation from 20 to 30% to within 10% [75]. For large-scale production, using automated mixing equipment to homogenize wheat straw of varying cellulose contents (35–45%) ensures consistent substrate quality, laying the foundation for stable industrial mushroom cultivation [119]. These technical optimizations collectively address key bottlenecks in agricultural waste utilization for edible mushroom cultivation. While each approach demonstrates significant benefits (e.g., improved yields, cost reduction, safety enhancement), their practical implementation must consider trade-offs such as scalability, resource availability, operational costs, and regional constraints. Future research should prioritize low-cost, decentralized solutions to enhance accessibility for diverse farming systems, alongside rigorous lifecycle assessments to quantify environmental and economic trade-offs.

**Table 4:** Benefits and limitations of strategies to address agricultural waste bottlenecks in edible mushroom cultivation.

Strategy Category	Specific Strategy	Benefits	Limitations	References
Alkaline Pretreatment	Using nematode (alkaline effluent from tortilla production) to soak agave bagasse	1. Reduces furfural content by up to 98%.	1. Scalability is limited to regions with maize-based food processing (reliance on nejayote as a tortilla industry byproduct).	[118]
		2. Boosts lignin degradation rate by 49%.	2. High alkalinity (pH > 11) may require neutralization to avoid substrate phytotoxicity for certain mushroom species.	
		3. Increases soluble sugar availability by 30–50%, creating ideal conditions for *Pleurotus ostreatus* and *Lentinula edodes* colonization.		
Physical Pretreatment	1. Crushing straw and banana leaf midribs to 2–5 cm fragments.	1. Enhances heat and moisture retention during composting.	1. Mechanical crushing increases energy consumption (2–3 kWh per 100 kg of biomass).	[75]
	2. Combining crushing with 121 °C steam sterilization for 30 min.	2. Accelerates *Pleurotus ostreatus* mycelial growth by 25–40% compared to untreated substrates.	2. Steam sterilization raises operational costs due to fuel/energy demands, infeasible for smallholder farmers in low-resource settings.	
Physical Pretreatment for Woody Wastes	Grinding hazelnut husk followed by steam treatment	1. Shortens mycelial colonization time by 3–5 days.	1. Requires specialized grinding equipment due to the hardness of woody materials (e.g., hazelnut husk), increasing upfront investment.	[59]
		2. Mitigates inhibition from high lignin content in woody wastes.	2. Efficacy varies with lignin type (syringyl vs. guaiacyl lignin), limiting universal applicability.	
Nutritional Regulation (Waste Blending)	Mixing rice straw with 10% poultry litter and 1% lime	1. Adjusts C/N ratio to the optimal 20–30:1 range.	1. Poultry litter may introduce pathogenic microbes (e.g., *Salmonella*) without proper composting, requiring additional safety checks.	[75]
		2. Increases Pleurotus ostreatus yield to 119 g per packet (42% higher than pure rice straw).	2. Odor from poultry litter is a nuisance in indoor cultivation facilities, needing mitigation measures.	
Nutritional Regulation (Waste Blending)	Mixing date palm leaves with wheat straw at a 25:75 ratio	1. Leverages wheat straw’s balanced nutrients to improve substrate palatability.	1. Wheat straw availability is seasonal and geographically constrained (limited in tropical regions).	[108]
		2. Raises biological efficiency by 18% compared to single-date palm leaf substrates.	2. Reduces reproducibility of the blend in diverse farming systems.	
Nutritional Regulation (Supplementation)	Adding corn meal as a supplement	1. Significantly enhances laccase activity in Agaricus bisporus.	No explicit limitations mentioned in the provided text.	[117]
		2. Promotes lignin decomposition by 22%.		
		3. Increases cumulative yield to over 34 kg/m^2^.		
Nutritional Regulation (Formulation Optimization)	Optimizing blend via Response Surface Methodology (12% rice bran + 25% food waste compost)	1. Boosts Pleurotus ostreatus yield to 91 g/bottle.	No explicit limitations mentioned in the provided text.	[101]
		2. Yield is 153% higher than the traditional formula (80% sawdust + 20% rice bran).		
Livestock Manure Treatment	Composting livestock manure at ≥60 °C for 7 days	1. Degrades over 90% of antibiotic residues.	1. Achieving and maintaining 60 °C uniformly in small-scale piles is challenging (poor insulation/irregular turning), leading to incomplete antibiotic degradation in some batches.	[68]
		2. High temperatures inactivate microbial communities involved in antibiotic persistence.	2. Long composting duration (≥7 days) delays substrate availability, impacting cultivation timelines.	
Coffee Ground Pretreatment	Pre-washing coffee grounds to remove excess phenolic compounds	Reduces inhibition of laccase activity by 30%, ensuring normal lignin degradation by *Pleurotus sajor-caju*.	No explicit limitations mentioned in the provided text.	[97]
Circular Utilization	Crushing spent mushroom substrate (SMS) and mixing it into new substrates at 20–40%	1. Replaces part of fresh lignocellulosic waste.	No explicit limitations mentioned in the provided text.	[105]
		2. Increases Pleurotus ostreatus biological efficiency by 15–20%.		
		3. Cuts raw material costs by 30%.		
Process Standardization	1. Controlling moisture at 65–70% via “hand-press test”.	1. Reduces batch yield variation from 20 to 30% to within 10%.	No explicit limitations mentioned in the provided text.	[75,119]
	2. Standardizing sterilization parameters (121 °C, 1.5 kg/cm^2^ for 1 h).	2. Ensures consistent substrate quality for large-scale production.		
	3. Using automated mixing equipment for wheat straw with varying cellulose contents (35–45%).	3. Lays the foundation for stable industrial mushroom cultivation.		

## 6. Outlook on Edible Fungi Utilizing Agricultural Wastes

As global attention turns to circular agriculture and waste reduction, edible fungi cultivation is emerging as a pivotal solution for agricultural waste valorization. Leveraging their unique ability to decompose lignocellulose, edible fungi not only convert diverse agricultural residues into high-value protein sources but also close nutrient loops in farming systems—aligning perfectly with the “Cycle Production of Plants, Animals, and Fungi” concept. The future of this field will revolve around four core pillars: localized resource utilization, technological innovation, circular model advancement, and standardized safety governance.

### 6.1. Localized Waste Utilization: Tailoring Solutions to Regional Endowments

The first priority is to maximize the potential of region-specific agricultural wastes, moving beyond one-size-fits-all approaches. In tropical regions, abundant wastes like banana leaves, cassava peels, and date palm fronds will be tapped into—for instance, mixing date palm leaves with wheat straw improves substrate palatability and boosts mushroom yields. Southeast Asia will focus on optimizing blends of sugarcane bagasse and rubber wood sawdust to replace pure sawdust substrates. In northern China, straw-manure mixtures will be the mainstay, while southern China will emphasize forestry waste utilization. For livestock-derived wastes like poultry litter, high-temperature composting will become standard practice to eliminate antibiotics, heavy metals, and pathogenic residues before they are used as nutrient supplements—ensuring both safety and nutritional balance.

### 6.2. Technological Innovation: Precision and Efficiency at the Core

Technological breakthroughs will center on efficient pretreatment and intelligent regulation to overcome traditional bottlenecks. Physically crushing straw to 2–5 cm fragments and combining it with steam sterilization will accelerate mycelial growth. For lignin-rich wastes like agave and tortilla residues, optimized alkaline pretreatment will enhance lignin degradation, making these underused resources viable for cultivation. Response surface methodology will refine substrate formulations—such as adjusting rice bran and food waste compost ratios—to significantly outperform conventional media. On the equipment front, intelligent systems with real-time sensors will monitor C/N ratios, humidity, and temperature during substrate preparation, while automated mixing gear will standardize quality and eliminate batch-to-batch variation. Gene editing will also play a role: enhancing fungi’s enzyme secretion capacity to boost lignocellulose degradation efficiency, and screening heavy metal-tolerant strains to ensure safety even on slightly contaminated substrates.

### 6.3. Circular Model Advancement: Closing Loops for Maximum Value

Circular utilization will be expanded to create a fully integrated waste-to-value chain. SMS will be a key focal point—crushing and mixing 20–40% SMS into new substrates cuts raw material costs by 30% and improves biological efficiency by 15–20%. SMS will also be processed into biochar or organic fertilizer, forming a “waste-substrate-fungi-SMS-fertilizer” loop that feeds back into crop farming. Additionally, combining mushroom cultivation with biogas production will recover both energy and nutrients from organic wastes, while new supplements like corn bran and gluten will enhance fungi’s lignocellulolytic enzyme activity—further promoting substrate degradation and yield.

### 6.4. Standardized Safety and Governance: Building Trust for Scaling

To support large-scale development, a robust system of standards and safety controls will be established. Classification standards for agricultural wastes, substrate quality evaluation systems, and edible fungi safety testing protocols will be unified to resolve inconsistencies from raw material variations. HACCP principles will be applied to compost processing to minimize contamination risks. Meanwhile, research into risk control technologies—such as biosorption to reduce pollutant residues—will be strengthened. These measures will not only ensure product safety but also lay the groundwork for industrial agglomeration.

The ultimate goal is to build integrated systems that link crop farming, livestock rearing, and fungi cultivation. This means promoting technology transfer to smallholders, developing region-specific waste formulations, and establishing regional linkage networks for waste collection, treatment, and mushroom production. By doing so, edible fungi cultivation will move beyond a standalone practice to become a linchpin of circular agriculture—reducing waste, recycling nutrients, and creating economic value. In turn, it will play a larger role in boosting food security, cutting carbon emissions, and advancing global environmental sustainability.

## 7. Conclusions

Agricultural waste, including animal-derived waste and spent mushroom substrate (SMS), offers opportunities and challenges as alternative substrates for edible fungus cultivation. Grounded in the “Cycle Production of Plants, Animals, and Fungi” theory, these waste materials can serve as nutrient sources for edible fungi, but their efficacy and safety depend on various factors. Nutritionally, while they provide essential carbon and nitrogen, precise regulation of the C/N ratio is vital; imbalances reduce biological efficiency, inhibit growth, and affect quality. Animal-derived waste may contain contaminants like antibiotics, heavy metals, and drug-resistant bacteria, threatening food safety and disrupting mineral balance. Physicochemically, traditional composting of animal-derived waste often fails to fully decompose, affecting pH stability, which directly impedes mycelial growth and indirectly reduces nutrient absorption. For both types of waste, controlling moisture is crucial: excess moisture impairs aeration and increases contamination risk, while insufficient moisture limits nutrient transport. SMS, despite its recyclable nutrients, has unstable nutrient content, fluctuating C/N ratios, variable pH, and inconsistent moisture, making it challenging to ensure uniform growth conditions for large-scale edible fungus cultivation.

Large-scale use of agricultural waste as edible fungus substrate presents four interconnected challenges, all of which are intricately related to critical substrate properties like nutrients, pH, and moisture. Lignocellulose-induced structural hurdles pose a significant issue: they slow down degradation, extend cultivation cycles, disrupt air circulation, and upset the moisture balance. A suboptimal structure leads to uneven moisture distribution—too much or too little moisture can hinder fungal respiration and nutrient absorption, and this can also indirectly affect pH stability. Nutrient and physicochemical imbalances are equally concerning. Nutritionally, imbalanced C/N ratios or irregular mineral nutrient levels can directly impede mycelial growth and fruiting body development, possibly even causing toxic reactions. Physicochemically, inappropriate pH levels can inhibit fungal growth by altering enzyme activity and microbial communities, reducing nutrient utilization. These imbalances collectively suppress enzymatic function and fungal proliferation. Contamination and anti-nutritional risks not only threaten food safety and reduce substrate value but also disrupt nutrient absorption, distort mineral balance, and affect pH stability, further exacerbating substrate unsuitability. Finally, batch instability and standardization challenges result in fluctuations in key properties such as nutrients, pH, and moisture, leading to inconsistent yields and higher production costs. For example, variations in moisture content from batch to batch can change contamination risks, while nutrient inconsistencies can impact product quality.

Targeted optimization strategies can enhance substrate quality through nutritional and physicochemical improvements: efficient pretreatment technologies disrupt lignocellulose structures, optimizing aeration, moisture balance, and indirectly pH stability; nutrient regulation via mixed application and supplementation precisely adjusts C/N ratios, supplies essential minerals, and directly boosts edible fungus yield and quality; tightened control over contaminants and anti-nutritional factors eliminates safety risks, ensures proper nutrient absorption, and prevents interference with pH and nutrient balance; promoting SMS recycling reduces costs and closes the resource loop, while process standardization strictly controls key properties like nutrients, pH, and moisture, guaranteeing consistent substrate quality and supporting the sustainable, large-scale growth of the edible fungus industry.

## Figures and Tables

**Table 1:** Collection of the number and proportion of studies in different periods.

Period	Number of Publications/Articles	Proportion of Publications/Articles
1978–1999	3	2.52%
2000–2009	14	11.76%
2010–2019	39	32.77%
2020–2025	63	52.95%
Total	119	100

**Table 2:** The C/N ratios of common agricultural waste materials.

Source	Substrate	C/N Ratio	Reference
Plant-Derived Wastes	Rice bran	20:1	[101]
	Wheat bran	22:1	[102]
	Wheat straw	203:1	[102]
	Agave bagasse	50:1	[103]
	Hazelnut husk	59:1	[59]
	Palm oil frond	125:1	[73]
	Cotton seed hulls	19:1	[74]
Animal-Derived Wastes	Dairy manure	24:1	[70]
	Pig manure	7:1	[104]
Microbial-Derived Waste	Spent shiitake mushroom substrate	50:1	[85]
	Spent *Pleurotus ostreatus* substrate	93:1	[102]
	Spent mushroom compost of Agaricus	12:1	[105]

**Table 3:** Comparative analysis of substrate composition, pretreatment methods, and yield performance in mushroom cultivation studies.

Cultivar	Substrate Composition (Ratios)	Substrate Pretreatment	Substrate Physicochemical Properties	Yield/Biological Efficiency	References
*Pleurotus ostreatus*	-DFT (15 mm corncob) -XFT (5 mm corncob) -84% corncob, 10% bran, 5% lime, 1% urea	-Chopped, soaked for 12 h -Sterilized at 121 °C for 1.5 h	-pH 10.0–11.0 -Moisture 60–70% -C/N ratio 33.13–36.95	-DFT: 55% BE -XFT: 70% BE	[34]
*Lentinula edodes*	-Hazelnut husk (HH) alone or mixed with: -Wheat straw (WS) -Beech wood-chip (BWC) -Wheat bran (WB)	-Air-dried, ground to 2–5 cm -Soaked in water for 12 h	-pH 6.2–6.91 -Moisture 60–65% -C/N ratio 48.82–117.82	-50 HH:50 WS: 105.1 g/packet -60 BWC:20 WS:20 WB: 233.92 g/kg	[59]
*Pleurotus ostreatus*	-Rice straw + 10% poultry litter + 1% lime -Banana leaf mid ribs + 10% cow dung + 1% lime	-Sun-dried for 10 days -Soaked in water overnight	-Moisture 65–70% -pH 10–11	-Rice straw + poultry litter: 119 g/packet -Banana leaf + cow dung: 116.7 g/packet	[75]
*P.ostreatus* *P. sajor-caju*	-Rice straw -Banana straw	-Ground into particles (2–5 cm) -Dried at 60 °C for 1 h	-pH 5.8–8.0 -Ash 5.36–5.86% -Protein 1.54–3.10%	-	[97]
*Pleurotus ostreatus*	-Cotton seed -Paper waste -Wheat straw -Sawdust	-Sun-dried for 10 days -Soaked in water for 12 h	-Moisture 65–70% -pH 7.0	-Cotton seed: 133.26% BE -Paper waste: 97.97% BE	[99]
*Pleurotus ostreatus* *P. eryngii* *Agaricus bisporus*	-Spent mushroom substrate (SMS) -Spent mushroom compost (SMC) -*Pleurotus* waste (PW) -*Agaricus* waste (AW)	-Pasteurized or sterilized -Supplemented with wheat bran/soybean flour	-pH 5.8–8.0 -C/N ratio 11.03–41.0	-*P. ostreatus*: 133% BE (SMS GZ-PW) -*P. eryngii*: 121% BE (WS-SMS GZ)	[105]
*Pleurotus ostreatus*	-Date palm leaves mixed with: -Wheat straw (WS) -Boobialla leaves (BL) -Sawdust (SD)	-Ground using grinder -Sterilized chemically with formaldehyde	-pH 7.0 -Moisture 60–65%	-25 DP:75 WS: 120 g total yield/bag -100 DP:0 WS: 8.2 g fruiting body/bag	[108]
*Coprinus comatus*	-Corn stalk	-Ground into 80-mesh -Sterilized at 121 °C for 1 h	-pH 5.0–5.3 -C/N ratio adjusted	-	[112]

## Data Availability

No new data were created or analyzed in this study. Data sharing is not applicable to this article.
